# Contextual factors and intentional rounding in acute hospitals: understanding what works, for whom, in what settings: a realist synthesis protocol

**DOI:** 10.12688/hrbopenres.13792.1

**Published:** 2023-12-01

**Authors:** Aileen Hetherton, Frances Horgan, Jan Sorensen, Siobhan E. Mc Carthy

**Affiliations:** 1 School of Postgraduate Studies, Royal College of Surgeons in Ireland, Dublin, Leinster, D02 YN77, Ireland; 2 School of Physiotherapy, Royal College of Surgeons in Ireland, Dublin, Leinster, D02 YN77, Ireland; 3 Health Outcomes Research Centre, Royal College of Surgeons in Ireland, Dublin, Leinster, D02 YN77, Ireland; 4 Graduate School of Healthcare Management, Royal College of Surgeons in Ireland, Dublin, Leinster, D02 YN77, Ireland

**Keywords:** Intentional Rounding, Nurse rounding, Patient rounding, Acute Hospital, Intervention, Falls Prevention, Realist synthesis

## Abstract

**Background:**

This study aims to understand and explain the influence of contextual factors on the implementation of Intentional Rounding in acute hospitals using the realist synthesis methodology.

Falls of hospital admitted patients are one of the most frequent concerns for patient safety in the acute hospital environment. The reasons why people fall are complex. International guidelines recommend a multifactorial assessment and effective prevention and management of identified risk factors in order to reduce the number of falls. One approach for delivering this is by Intentional Rounding (IR). IR is an umbrella term, understood as a structured process whereby nurses or care staff carry out regular checks with individual patients using a standardised protocol to address such issues as positioning, pain, personal needs and placement of possessions.

**Methods:**

This study will use realist synthesis to understand what works, for whom, in what circumstances and in what settings. Realist synthesis is a theory driven interpretive approach to evidence synthesis. It is the intention of the researchers to analyse IR as an intervention, which aims to enhance patient care and safety in hospital settings. The synthesis forms part of a larger implementation study examining interventions that reduce the number of falls that occur in hospitals. Search terms will include intentional rounding, purposeful rounding, comfort rounding and hourly rounding and will encompass search terms beyond IR and falls rates in order not to limit the synthesis. This synthesis will conform to the RAMESES (realist and meta-narrative evidence synthesis group) publication and reporting quality standards for a realist synthesis.

**Conclusions:**

The findings will inform the next phase of an implementation study on IR in acute hospital settings, namely selection of an IR approach and evidence informed barriers and enablers to its implementation. The results will be disseminated in a peer-reviewed journal and through presentations.

## Introduction

Falls pose a significant challenge to healthcare systems internationally, with ageing populations and increasingly higher expectations of active living ( Gerrish *et al*., 2019). Falls are a leading cause of non-fatal injuries among older adults and these injuries may have physical, social and psychological effects. In Ireland, hospital falls are the most commonly reported incident within the Health Service Executive (HSE) with 34,114 falls being reported in 2021. Over half (n= 18,023) of these occurred in acute hospitals ( HSE, 2022). Falls are costly to patients, carers and society from a physical, psychological and economic perspective. Falls lead to physical harm, prolonged hospital stays, decreased quality of life for patients, and affects those who care for them and society in general ( WHO, 2021). 

A fall is defined as an event, which results in a person coming to rest inadvertently on the ground or floor or other lower level ( WHO, 2021). There are different types of falls - assisted, injurious, negligible or no harm and the reasons why they occur are complex and multifactorial ( State Claims Agency, SCA, 2019). Intrinsic risk factors are related to the person, including age, history of falls, physical illness, cognitive impairment, medications and movement disorders. Extrinsic risk factors include elements of the environment: lighting, flooring, clutter, unstable furniture and faulty walking aids ( NICE, 2013).

The cost of falls worldwide is in excess of €400 billion each year ( WHO, 2021). There is speculation of continued rising costs across countries yet data on falls incidence with associated costs are not collated routinely in countries. In Ireland, fall-related injuries in older people cost at least €402 million to the Irish economy ( HSE, 2008). A National Fall Prevention Strategy has yet to be developed. In the US, using 2015 data, falls cost the economy an estimated $50 billion per year ( Florence *et al.*, 2018) and falls and fall related fractures cost the National Health Service (NHS) more than £2.3 billion per year in 2013. However, these figures are not current and much has changed since these data were collected. The costs of falls in healthcare are understudied and better and routine measurement is needed to ensure that falls-prevention strategies are economically sound. The World Health Organisation ( WHO, 2021) estimate that 37.3 million falls require medical attention per year (2017–2020), with an estimated 684,000 individuals dying because of a fall annually worldwide ( WHO, 2021). 

To reduce the number of falls that occur in hospital settings, international guidelines recommend a multifactorial assessment and the cost-effective prevention and management of identified risk factors ( NICE, 2013). Multifactorial interventions are recommended in the literature as a mechanism to prevent in-hospital falls ( AHRQ, 2013). Individualised interventions expounded from assessment include hypotension treatment, footwear assessment and mobility treatment, among others. However, these individual interventions will assist a limited cohort of patients. Systematic approaches target a wider group of patients to help reduce inpatient falls on a large scale. IR is an example of a systematic approach and aims to complement stand-alone interventions.

Initially known as “hourly rounding” ( Meade *et al.*, 2006), IR is an umbrella term, understood as a structured process whereby nurses or care staff carry out regular checks with individual patients using a standardised protocol to address such issues as positioning, pain, personal needs and placement of possessions ( Harris *et al.*, 2017). In 2006, a landmark US study developed a specific intervention recognised as IR and examined its effects on patient satisfaction, call bell use and patient falls. There are many terms used for this process, including “comfort rounding”, “care rounding”, “purposeful rounding”, “patient rounding” and “nursing rounding” among others ( Sims *et al.*, 2018). 

In Ireland, the adoption and use of IR has yet to be formally studied. In the UK, IR came to favour following recommendations from the Mid Staffordshire report to have systematised and regular interaction and engagement between nurses and patients on ward rounds ( Mid Staffordshire NHS Foundation Trust Public Inquiry (2013, Vol III). Although IR was not explicitly recommended, the concept of regular rounding on patients underpinned the recommendations made. The British Government endorsed IR in 2012 and advocated IR as a method of proactively addressing patients’ needs. As the NHS introduced IR across trusts, some limitations became evident; for example, staff engagement was limited in areas where education programmes had not preceded the introduction of IR ( Sims *et al.*, 2018). 

While there are established benefits of IR, including improved nurse-patient communication and improved visibility of nursing staff, there are a number of methodological limitations in the IR studies, such as bias, weak study design and conflicts of interest have been identified ( Snelling, 2013). There is also little information available on the implementation costs and value-for-money of IR, which is important to inform what IR protocol is selected and how it is implemented ( OECD, 2022).

There is an information gap on the ‘how’ and ‘why’ and in ‘what context’, IR works. This realist synthesis forms the first phase of a study and will examine the existing evidence on IR and develop a programme theory of how IR works in an acute hospital setting, with whom and in what circumstances. Using the programme theory, the second phase of the study will involve the design, testing, implementation and evaluation of an IR intervention to reduce inpatient falls in a hospital setting. Results from the synthesis will inform the design and evaluation of strategies to address the barriers and enablers of implementation of IR in acute hospital settings.

### Rationale and existing reviews

Research on IR has focussed on the effectiveness of IR on call bell usage, patient satisfaction rates, falls, pressure ulcers and pain management ( Meade *et al,*, 2006) ( Lucas, 2010). Results from the Meade study found that IR reduced call bell use and falls and improved patient satisfaction rates when completed hourly, but the acknowledged use of quasi-experimental approaches may have produced more positive results than a random assignment. Based on staff reporting, Lucas found that IR reduced pressure ulcer incidences and improved pain management. The results of these studies suggest that further research on the implementation and potential effects of IR on patient outcomes is required. There is a dearth of research focussing on the barriers and enablers to implementation of IR. IR has been the focus of literature reviews who found that it has mixed results, and contextual factors play a part in its success or otherwise ( Harris *et al.*, 2017; Ryan *et al.*, 2019). A recent systematic mixed method review that included twenty-one studies, found that evidence is mixed and quality of data is not robust, due to weak methodological design of many of the studies and a large variation in sample sizes ( Christiansen *et al*., 2018). Ryan *et al.* (2019) conducted an integrative literature review of IR and found that overall it is a positive intervention as it encourages improved nurse patient interaction. They recommend further research around how it has been implemented, what was working well about IR, what the barriers are and in what context it is successful.

A small number of existing literature reviews demonstrate the contextual factors influencing IR ( Christiansen *et al.*, 2018; Sims *et al.*, 2018). These offer explanations about how it works well in some areas where patients may need regular interaction e.g. care of the older person units, and may not be successful in other areas, e.g. a busy surgical ward where the patient is mostly independent ( Christiansen *et al.*, 2018). There has been only one realist review completed on fall prevention programs using IR ( Sims *et al.*, 2018). There has been no study in Ireland on the implementation of IR in an acute setting and how it may impact on falls. This review, as part of a larger implementation study on IR in Irish hospitals, is necessary to learn more about what it is about IR that enhances patient safety and prevents harmful outcomes for patients.

The question about IR, and for whom it will work, in what circumstances and how it works, is best answered using a realist synthesis, as there is a requirement to understand more fully how and for whom IR is successful in an acute hospital setting. Duddy and Wong (2023) recommend that a realist approach is best suited when the outcome of an intervention is likely to be variable and context dependent. A realist synthesis will assist in the development of a programme theory about why IR works in some areas and not in others based on learning from previous studies. As this synthesis is part of a larger implementation study, searching only evidence related to IR and falls rates would limit the synthesis. Therefore, it is our intention to analyse IR as an intervention, which affects patient care and safety in hospital settings.

Sims *et al.* (2018) carried out a realist synthesis of IR in acute hospital wards that informed an evaluation project ( Harris *et al.*, 2019). The literature search spanned from 2006 up to and including 2016, 44 papers were reviewed spanning a variety of settings such as accident and emergency, orthopaedic, maternity, medical and surgical units, mental health and intensive care. Harris *et al.* (2019) discussed how previous studies failed to explain how IR works in a specific context and what drives IR to succeed or otherwise. They found that IR reduces potential harm when implemented in a comprehensive and consistent manner but that there is limited evidence of how it works in practice. They concluded that a poor understanding of how IR works poses a major challenge to learning, replication and sustainability of the intervention. 

## Realist synthesis methods

Realist synthesis is a theory-driven approach to evaluation. It enables the researcher to answer the question “what works for whom under what circumstances, how and why?” ( Wong *et al.*, 2013b). A realist synthesis may begin with an initial search of literature to illicit how an intervention is avowed to work (the initial programme theory). It is suited to complex interventions such as IR as any intervention that is used and adapted in healthcare is prone to modification due to many variables such as staff engagement and perception, environment, education, policy and patient factors among others. IR is a good example of a complex social intervention as it is multicomponent and the outcomes and resource implications may be influenced by the context in which it occurs. 

A realist synthesis approach views causation as generative: mechanisms might be triggered within certain contexts resulting in one or more outcomes following an intervention ( Pawson *et al.*, 2005). There is no simple ‘yes’ or ‘no’ answer to the question of the implementation and cost effectiveness of IR unless we explore what makes it successful, for what type of patient and in what circumstances. The identification and understanding of context-mechanism-outcome configurations may achieve this. The realist synthesis approach is required to help policy-makers and healthcare professionals to understand how IR may alter contexts, which then trigger hidden mechanisms that reduce falls and produce other intended or unintended outcomes.

In order to identify and describe the resource use and cost requirements of the implementation of IR in the hospital setting, the use of the realist synthesis methodology is not the most obvious choice, as most researchers would look to an economic evaluation to assess cost-effectiveness of an intervention and implementation strategy ( Anderson & Hardwick, 2016). Realist evaluation will typically explain how an intervention works, in what circumstances and why, but in order for decision-makers and service managers to accept an intervention as financially viable, it should be affordable and cost-effective. In order to compare costs and the impacts of an intervention, decision-makers often use economic evaluations as appraisal tools. However, economic evaluations do not often consider context and the results are difficult to generalise to other areas and so for this reason a combination of both realist and economic evaluation may assist in this study involving a complex intervention. Coast *et al.* (2000) found this following collaboration between economic researchers in the UK. They suggested that context, capacity and the timing of a study might affect its acceptance by decision-makers and practitioners.

Anderson and Hardwick (2016) proposed that cost effectiveness should be articulated in realist synthesis. The relation between costs and outcomes, and in favour of selecting interventions and implementation strategies that are cost effective can inform the programme theory. In order to calculate costs of an initiative, the cost of harm is a factor. With in-hospital falls, there are direct costs such as longer length of stay, diagnostics, surgery, health professional wages etc. The unknown additional costs that need to be considered include follow-up care in a non-acute setting, consequent hospital readmissions and outpatient appointments, a loss of wages for the individual or their family carers, among others. The principles of economic evaluation, i.e. resource use and responses to resource use will form part of the criteria for this synthesis. There are no economic evaluations on the implementation of IR in an Irish hospital setting so the advancement of knowledge on IR and whether it is a viable option to consider is necessary and timely.

### Research aim and objectives

***Research aim***

This study aims to understand how, when and under what circumstances does IR in acute hospital settings promote stakeholder engagement in IR and improve patient safety outcomes.

***Objectives***

1. Determine how IR has been implemented in hospital settings.
2. Develop a range of programme theories that describe how IR works, for what type of patient, in what setting and with what types of staff, in what circumstances and why.
3. Identify and describe the most important mechanisms by which IR is thought to produce better outcomes for patients, healthcare staff and the service in the hospital setting.
4. Identify and describe the contextual factors that enable or block the impact of these mechanisms (enablers and barriers)
5. Identify and describe the resource use and cost requirements or impacts of the different mechanisms and contextual factors related to developing and implementing IR in a hospital ward
6. Synthesise the data using realist methodology to explain the circumstances in which IR is likely to be effective and cost effective in a hospital setting.

## Methods

This synthesis will conform to the RAMESES (realist and meta-narrative evidence synthesis group) publication and reporting quality standards for a realist synthesis ( Wong *et al.*, 2013b; Wong *et al.*, 2016). Realist synthesis is a theory-driven approach designed for evaluating complex social interventions such as IR ( Sims *et al.*, 2018). Realist inquiry postulates that the outcomes of an intervention are influenced by how it is implemented and in what context it is conveyed ( Pawson *et al.*, 2004). A key output of a realist synthesis is a programme theory or theories and understanding the success or failure of an intervention by asking exploratory questions. These questions include: What is it about this intervention that works? For whom? In what circumstances? These questions may be answered through the identification of context-mechanism-outcome configurations ( Sims *et al.*, 2018).

This synthesis will be based on the stages of a realist review put forward by Pawson *et al.* (2004) with the addition of a further stage of stakeholder involvement for theory refinement, which was added by Molnar *et al.* (2015) and used by Power *et al.* (2019) see box 2.

**Box 2. Planned Stages of Realist Synthesis ( Hunter *et al*., 2022) ( Pawson *et al*., 2004)**

**1. Identify the Review Question**

    a.  Map the research area

    b.  Informal literature search

    c.  Concept mining

    d.  Develop initial rough programme theories

    e.  Consult stakeholders

    f.  Build hypothetical model of key initial rough programme theories to test in literature

**2. Searching for primary studies**

    a.  Database search

    b.  Skimming for relevance – Citation tracking

    c.  Snowballing

    d.  Grey literature

    e.  Contact authors

**3. Quality Appraisal**

    a.  Bespoke screening and appraisal tools

    b.  Consider rigour and relevance

**4. Extracting the data**

    a.  Extraction templates

    b.  Rich description and thick detail

**5. Synthesising the data**

    a.  Juxtaposing

    b.  Adjudicating

    c.  Reconciling

    d.  Consolidating

    e.  Situating

**6. Disseminating the findings**

    a.  Consult with stakeholders for widest impact

    b.  Recommendations based on middle range theories around which particular aspects of programme implementation need to be considered

Publication

### Stage one: clarify scope and developing an initial programme theory

In the initial stage of the synthesis, a literature search will be carried out to locate existing theories that explain how IR works, in what circumstances, for whom and how it generates outcomes for patients. To conduct a realist synthesis, it is important that a broad range of empirical research is included. The realist synthesis method, as described by Pawson *et al*. (2005), highlights that a significant amount of papers will be identified through "snowballing", that is using the reference lists of relevant articles to identify further papers for review.

Sims *et al.* (2018) conducted a realist synthesis of IR and used seven terms to describe the concept of IR for their literature search and this study will use the same strategy. This synthesis will consist of two phases; Phase One is theory-initiation and development where a programme theory of IR will aim to explain how and why IR is thought to generate outcomes for patients. Phase two will involve theory testing, where the programme theory will be reviewed and rival explanations to the theory developed will be explored. The realist synthesis is part of an implementation study, testing IR in a variety of acute ward settings in hospitals in order to assess whether IR is an effective and cost-effective intervention in reducing falls in the acute hospital setting.

In order to develop programme theories, an initial immersion in sources of information about the effectiveness and cost-effectiveness of IR will be completed. The exclusion/inclusion criteria are necessarily broad in order to ensure the theory development phase is able to take in the widest range of evidence of theories ( Hardwick *et al.*, 2013). A preliminary informal search (stage one) of literature was conducted in CINAHL and PubMed in November 2022. The informal searches conducted in stage one are different from the more formal searching that will be conducted in stage two, as they are exploratory in nature. Programme theory development will necessitate iterative discussions within the research team to progress competing theories in to an initial programme theory ( McConnell *et al.*, 2022). 

### Stage two: search strategy

The search strategy has been planned with the assistance of an Information Specialist. The major healthcare databases and repositories will be searched. See Box 1 for a list of search terms and databases that will be used for the search strategy. There will necessarily be further literature searches based on analysis of the preliminary evidence. Realist synthesis are iterative in process and there will be ongoing need for searching of databases throughout the synthesis until saturation is reached ( Pawson *et al.*, 2004). The references and citations in the 2018 realist synthesis and 2019 evaluation papers on IR in the hospital setting by Sims *et al.,* and Harris *et al.,* respectively will be used as a starting point for review of the literature and theory development.

**Box 1. Search Strategy**

**Databases that will be utilised for search include**

CINAHL, Medline/PubMed, PsycINFO, Embase, Cochrane Library and any other relevant databases identified by the information specialist

**Search terms to be used for Intentional Rounding**

Intentional round OR hourly round OR patient round OR comfort round OR purposeful round

OR proactive nurse round AND acute hospital setting or hospital or acute ward

The inclusion and exclusion criteria will be broad in order to ensure that the theory development phase will take in the widest range of evidence of theories. In line with Sims *et al.* (2018) criteria, areas of general weakness in evidence and individual study weakness will be reported where appropriate.

**Inclusion criteria:**

➢ Studies on implementation of IR in acute hospital settings.
➢ Population will include inpatients over 18 years of age in hospital
➢ Study design type includes all types of design which will help to direct theory development
➢ Any publication deemed relevant by the researcher (i.e. opinion pieces in peer-reviewed journals, healthcare policy documents, studies including other interventions to reduce falls in acute hospitals that may add to theory development

**Exclusion criteria:**

➢ Studies not in the English language.
➢ Studies in non-acute hospital or community settings.

### Stage three: selection and appraisal

During the initial phase of the synthesis, sources that contribute to theory development will be reviewed through a process of note taking, annotation and conceptualisation. The abstracts of all papers identified by searches will be screened for suitability by AH. Allocation of abstracts to other members and double screening of all abstracts by another member of the team will ensure rigour. The entire team will discuss conflicts until agreement is reached. Potentially relevant papers will be assessed using a structured data extraction form developed by AH. Inclusion criteria are studies (a) conducted in acute hospitals, and (b) implemented and tested a version of IR as a single intervention or allied to/bundled with other existing interventions. The relevance and contribution of documents to theory building will be part of the selection criteria.

After relevance checks, the full text of the literature will then be retrieved and independently assessed for rigour by AH. This is in line with RAMESES publication and reporting standards for realist synthesis as described by Wong *et al.* ( 2013a; 2016). Further searching will arise due to the iterative fashion of the review and the team will decide selection criteria based on whether the literature can further refine theory ( Power *et al.*, 2019).

For the different study types, a range of quality assessment tools will be used as appropriate to the study design, for example, the Joanna Briggs Institute (JBI) critical appraisal tools. These appraisal tools assess the methodological quality of a study and determine the extent to which a study has addressed the possibility of bias in its design, conduct and analysis. Any disagreements over eligibility based on quality will be resolved through discussion by the team.

### Stage four: data extraction

The included data will be uploaded to an Excel spreadsheet to include variables summarised in the Table 1 below. The Standards for Reporting Implementation Studies (StaRI) Checklist by Pinnock *et al.* (2017) has been used to guide this extraction tool.

**Table 1.** Data Extraction Tool - Adapted from the Standards for Reporting Implementation Studies (StaRI) Checklist ( Pinnock *et al.*, 2017).

Checklist Item	Variable	Details of data to be retrieved
1	Author and Year	
2	Title	
3	Rationale for study	The scientific background and rationale for the implementation strategy (including any underpinning theory/framework/model, how it is expected to achieve its effects and any pilot work.
4	Details of intervention	Including evidence about its effectiveness and how it is expected to achieve its effects
5	Country of Origin	
6	Aims and Objectives	The aims of the study, differentiating between implementation objectives and any intervention objective
7	Methods – Design and Key features	Cross referencing to any appropriate methodology reporting standards
8	Details of Intervention	Include details of intervention, frequency, who completed it, what it consists of, i.e. what needs it addressed for the patient
9	Details of implementation strategy	How was it carried out – education, role modelling, protocol,
10	Context/Setting	The context in which the intervention was implemented (consider social, economic, policy, healthcare, organisational barriers and facilitators that might influence implementation elsewhere).
11	Details of the site chosen for implementation	Location, specialty, personnel, resources – single site or multi-site
12	Sample size	Include number of patients the intervention was tested on. Rationale for sample size?
13	Duration of data collection	
14	Evaluation	Outcomes – Include Organisation, Staff, Patient, Process or implementation outcomes – what was expected and what happened
15	Results	Outcomes
		Characteristics of population
		Process Outcomes
16	Resource Use, costs, economic outcomes	Is this reported? Are they once-off costs, implementation costs, cost-outcome descriptions/relationships?
17	Implementation and Intervention Fidelity	Did the implementation strategy go as planned and was the intervention delivered as planned
18	Methods of analysis	How did they analyse the findings?
19	Context	The backdrop of programmes – triggers or modifies the behaviour of a mechanism
20	Mechanism	An underlying entity, process or structure that operates in a particular context to generate an outcome
21	Outcomes	The result of interaction between a mechanism and its triggering context
22	CMO Configuration	How a context triggers or changes the behaviour of mechanisms (enables or blocks) and produces an outcome

Contexts, Mechanisms and Outcomes will be extracted from papers. This synthesis will take consideration of the programme theories that Sims *et al.* (2018) explored for IR and use these to guide new theory development. In line with the RAMESES realist synthesis guidance by Wong *et al.* (2013b), “all theories remain theories that can be refined or disproved as new evidence comes to light”. Literature from 2016 to 2022 will be reviewed using the same search criteria and databases for identification of any new mechanisms of IR that may be relevant. Data from each of the studies selected will be analysed thematically to provide a comprehensive description of the purported mechanism of IR. Contexts that appear to activate or inhibit the mechanisms will be identified and outcomes when the mechanism is present or absent will be noted ( Harris *et al.*, 2017). A realist causal explanation for an outcome involves the researcher understanding the mechanism that produces the outcome and identification of what is functioning as context to activate the mechanism ( Duddy & Wong, 2023). Text related to context, mechanisms and their relationship to outcomes will be coded by AH. The codes used will be inductive (created to categorise data reported in included studies), deductive (created in advance of data extraction based on the initial programme theory) and retroductive (created based on interpretation of the data to infer what the hidden causes might be for outcomes). This process is similar to that being used by Ford *et al.* (2021) in their EQUALISE study on health inequalities.

### Stage five: data synthesis and analysis

The key analytic process in a realist synthesis involves iterative testing and refinement of theoretically based explanations (programme theories) using empirical evidence from data sources ( Wong *et al.*, 2013b). Programme theories will be tested and refined. This will be achieved by drawing comparisons with the evidence, exploring, and analysing the relationships between contexts, mechanisms and outcomes. Evidence will be compared to identify recurring patterns of CMOs across the data including trends that may support, contradict or generally inform the programme theory. Iterative analysis will be carried out to refine the identified CMO configurations in order to fully explain how, why and in what circumstances IR may successfully or otherwise be implemented ( Coles *et al.*, 2017; Power *et al.*, 2019).

Rayan software will assist in management of the data and a thematic analysis approach will be adopted to synthesise findings. All included studies will be analysed by AH. The process of synthesis will include: (1) comparison of findings from different studies; (2) using findings from studies to address the purpose(s) of the synthesis; (3) seeking both confirmatory and contradictory findings; (4) refining programme theories in the light of evidence; and (5) disseminating the review with findings, conclusions and recommendations. Data analysis should take a “retroductive” approach – using both inductive and deductive approaches to identify hidden or causal factors that lie beneath patterns of change. The researcher must test and refine programme theories ( RAMESES II project, 2017). The realist synthesis will include diverse sources of evidence and synthesis structured around the analytical activities described in Pawson *et al.* (2004), including juxtaposition, reconciliation, adjudication, consolidation and situation of evidence. These activities when clearly documented in a realist review achieve transparency of synthesis ( Hardwick *et al.*, 2013).

### Stage six: theory refinement with stakeholders

A stakeholder consultation workshop is planned following on from data synthesis, in order to refine the programme theory. The group will consist of people involved directly in patient care, management, quality, finance and patient or family representatives. This workshop will serve two purposes. It will aid the process of theory development and refinement and ensure that any research findings are clear and useful to those involved in providing and receiving IR in a hospital setting. Brennan *et al.* (2014) describe this process as a reality check, to assist in validation of the programme theory for use in clinical practice. This process is recommended in realist synthesis as understanding what key stakeholders know about an intervention and their reasoning for or against its implementation is essential to understanding it ( Harris *et al.*, 2017).

## Dissemination of findings

Findings and results from this synthesis will be disseminated and shared with stakeholders, frontline practitioners and policy makers. They will be shared as a final report with presentations to stakeholders and practitioners. The results will be utilised to inform the next stage of a larger implementation study on the effect and cost-effectiveness of the implementation of IR in acute hospital settings on fall rates. The work will be presented at a relevant national conference, and publications from the review will be written up and published in a peer reviewed academic journal.

## Discussion

This study will use a realist synthesis approach to synthesise the available evidence and enable a better understanding of what works, for whom and in what circumstances, when, how and why in relation to IR in acute hospital settings. It is intended to build on the most recent realist synthesis on IR, which was published in 2018. The use of a realist approach will allow the synthesis to describe and explain how and why IR works in different contexts by exploring programme theories and the interactions between contexts, mechanisms of change and outcomes.

The emphasis in this synthesis on context-sensitive findings will offer broad principles that may be applied in different situations and circumstances ( Gordon *et al.*, 2020). The chosen approach will ensure that current evidence of contextual enablers and barriers can be taken in to account in designing and planning the implementation phase of a larger project.

## Data Availability

All data underlying the results are available as part of the article and no additional source data are required.
