# Supplementary material for: Contextual factors and intentional rounding in acute hospitals: understanding what works, for whom, in what settings: a realist synthesis protocol
Source: HRB Open Res. 2024 Aug 21;6:72. Originally published 2023 Dec 1. [Version 3] doi: 10.12688/hrbopenres.13792.3 (PMC11234086; doi:10.12688/hrbopenres.13792.3)
Supplement: Peer review history

# Reviewer report: Reviewer response for version 3

**DOI:** 10.21956/hrbopenres.15324.r43677

**Published:** 2024-12-20

**Authors:** Janet Long

**Affiliations:** 1 Macquarie University, Sydney, Australia

This is a protocol for a realist synthesis of intentional rounding as an intervention to address falls in hospitals. Falls have been a costly adverse event (in terms of human suffering, as well as money and resources) that have persisted in spite of decades of targeted interventions. Building on previous work that is now dated, they will determine what contextual factors are associated with desired outcomes. References are current and authoritative in this field showing their firm understanding of realist methodologies. I have noted the previous comments and responses. I think the protocol is acceptable and good quality. I wish them all the best in the execution of the study.

Is the study design appropriate for the research question?

Yes

Is the rationale for, and objectives of, the study clearly described?

Yes

Are sufficient details of the methods provided to allow replication by others?

Yes

Are the datasets clearly presented in a useable and accessible format?

Not applicable

Reviewer Expertise:

Health systems research, Implementation science, knowledge translation, realist evaluation

I confirm that I have read this submission and believe that I have an appropriate level of expertise to confirm that it is of an acceptable scientific standard.

# Response

**Published:** 2024-12-20

**Authors:** Aileen Hetherton

**Affiliations:** School of Post Graduate Studie, Royal College of Surgeons in Ireland Faculty of Sports and Exercise Medicine, Dublin, Leinster, Ireland

Thank you Janet Long for your review of this protocol. 

 Your comments were very helpful as we continue our work on falls prevention interventions. 

 Regards

 Aileen Hetherton

# Reviewer report: Reviewer response for version 3

**DOI:** 10.21956/hrbopenres.15324.r42498

**Published:** 2024-10-10

**Authors:** Alice Hemenway

**Affiliations:** 1 University of Illinois at Chicago, Chicago, USA

The article describes a planned review of intentional rounding, focusing on what works for whom, in what certain circumstances, and in what settings. They describe the plans for a realist synthesis process in 6 stages (identify the question, search, quality appraisal, data extraction, synthesizing and dissemination). The article provides a level of detail of the methodology to allow replication.

Is the study design appropriate for the research question?

Yes

Is the rationale for, and objectives of, the study clearly described?

Yes

Are sufficient details of the methods provided to allow replication by others?

Yes

Are the datasets clearly presented in a useable and accessible format?

Not applicable

Reviewer Expertise:

Internal medicine, use of theory in pharmacy services

I confirm that I have read this submission and believe that I have an appropriate level of expertise to confirm that it is of an acceptable scientific standard.

# Reviewer report: Reviewer response for version 2

**DOI:** 10.21956/hrbopenres.15239.r40811

**Published:** 2024-07-22

**Authors:** Ruth Harris, Sarah Sims, Mary Leamy

**Affiliations:** 1 King's College London, London, England, UK; 2 King's College London (Ringgold ID: 4616), King's College London, London, UK

We have reviewed the revisions and it appears that the authors have not responded to our comments. Therefore, we are unable to amend our previous status of 'Not Approved.' We would be happy to review the paper when they address our comments.

Is the study design appropriate for the research question?

Yes

Is the rationale for, and objectives of, the study clearly described?

Partly

Are sufficient details of the methods provided to allow replication by others?

No

Are the datasets clearly presented in a useable and accessible format?

Not applicable

Reviewer Expertise:

NA

We confirm that we have read this submission and believe that we have an appropriate level of expertise to state that we do not consider it to be of an acceptable scientific standard, for reasons outlined above.

# Response

**Published:** 2024-08-15

**Authors:** Aileen Hetherton

**Affiliations:** School of Post Graduate Studie, Royal College of Surgeons in Ireland Faculty of Sports and Exercise Medicine, Dublin, Leinster, Ireland

The realist synthesis protocol has kindly been reviewed by our colleagues in King’s College, London. Professor Ruth Harris, Sarah Sims and Dr Mary Leamy have provided valuable commentary and feedback to enable improvements in this protocol.

 There has been a revision of the section regarding the origins of Intentional Rounding (IR) in the USA by the Studer Group in 2007. The reference list and paper has been updated with two more references including a more recent publication by Leamy et al., (2023),  which has been published since version 1 of this protocol.  This paper has provided greater focus for the proposed realist synthesis. The rationale for this review has been updated to reflect how this realist synthesis is intended to further refine the programme theory which was published by Leamy et al (2023).  The two programme theories which were supported in the evaluation in hospital ward settings have been a starting point for the current realist synthesis and have provided valuable insight into the implementation of IR at the frontline in hospital wards. There has been 32 more international research studies on intentional rounding in acute care settings since the literature search prior to publication of the realist evaluation in 2019 and it is intended to incorporate this research into the current synthesis to build on and refine the programme theory. The methods section has also been updated based on the valuable commentary.

# Reviewer report: Reviewer response for version 2

**DOI:** 10.21956/hrbopenres.15239.r40810

**Published:** 2024-07-09

**Authors:** Amy Drahota, Leah Fullegar

**Affiliations:** 1 University of Portsmouth, Portsmouth, England, UK

Thank you for considering our previous feedback. We have no further comments to make.

Is the study design appropriate for the research question?

Yes

Is the rationale for, and objectives of, the study clearly described?

Partly

Are sufficient details of the methods provided to allow replication by others?

Partly

Are the datasets clearly presented in a useable and accessible format?

Not applicable

Reviewer Expertise:

falls prevention; health & care of older adults; systematic reviews; realist synthesis.

We confirm that we have read this submission and believe that we have an appropriate level of expertise to confirm that it is of an acceptable scientific standard.

# Reviewer report: Reviewer response for version 1

**DOI:** 10.21956/hrbopenres.15090.r38252

**Published:** 2024-04-12

**Authors:** Ruth Harris, Sarah Sims, Mary Leamy

**Affiliations:** 1 King's College London, London, England, UK; 2 King's College London (Ringgold ID: 4616), King's College London, London, UK

Thank you very much for asking us to review this paper reporting the protocol for a realist synthesis of intentional rounding. First of all, we must acknowledge that we are members of the team that undertook the NIHR funded realist synthesis and evaluation that this paper refers to (Harris et al 2017, 2019, Sims et al 2018). We are very pleased that another research team is planning to build on our work and to test out and refine further the programme theories that we identified in our realist synthesis and tested in the realist evaluation of intentional rounding in hospital wards in England. We have a number of questions and comments which we hope will be helpful to authors as they develop their work:

*Introduction*

- The introduction to the proposal focuses the review on intentional rounding as a multifactorial intervention to reduce falls.  Although this is an assumption made in many published papers, the research evidence for this is very weak due to serious methodological and/or reporting weaknesses. In most healthcare organisations there are several interventions which are implemented to reduce falls, e.g., falls assessments, enhanced care, bay tagging, medicine review etc., and so it would be very difficult to identify the impact of intentional rounding. Furthermore, no evidence was found to support this in our evaluation of intentional rounding. *There is a need to provide a clear rationale to focus on falls and not other outcomes (intended or unintended).*
- It would be useful to say more about the intentional rounding intervention **, **its origins and the reasons for adoption. For example, there is no mention of the Studer group and there is no reference for the "landmark US study in 2006" that is referred to.
- Given that there is an existing realist synthesis it would be helpful to provide an idea of how many new papers about intentional rounding have been published since 2016 to provide a rationale for the need to repeat/update it.
- The realist synthesis is presented as the preparation for a realist evaluation study in acute hospital settings in Ireland. It is stated that there is an evidence gap in how, why and in what context IR works and that it is necessary to learn more about what it is about IR that enhances patient safety and prevents harmful outcomes for patients. However, although the previous realist synthesis and evaluation are frequently cited, this protocol paper doesn’t report the findings of either or provide details of how this proposed realist synthesis builds on the programme theory identified in the previous review and tested in the realist evaluation study. In our realist evaluation on acute hospital wards in England we found little or no evidence that the intervention works in the way it was expected to work or had an impact on patient safety.  
- It is not completely clear, but it appears that the plan is to repeat the realist review to generate a programme theory rather than update/refine the existing programme theory. We think it would be more useful to continue refining the programme theory of intentional rounding from the current empirically informed programme theory. We have a recent paper which provides more detail of the realist evaluation which may be useful (Leamy et al 2023) [Refer ref 1]

*Methods*

- Stage one: clarifying scope and developing initial programme theory – the authors state that they are undertaking a realist to identify existing theories of IR but as mentioned above they do not share the programme theories that have already identified in the previous synthesis. They state the *‘the references and citations in the 2018 realist synthesis and 2019 evaluation papers on IR in the hospital setting by Sims et al., and Harris et al., respectively will be used as a starting point for review of the literature and theory development*.’ This suggests that they plan to start the review from scratch rather than starting from existing realist programme theories and searching for new evidence to support or refute them.  
- Use of the Joanna Briggs Institute critical appraisal tools – It would be useful to provide a rationale for the use of these tools. In realist approaches, the quality of evidence is usually assessed a different way focussing on the quality of evidence on refining understanding of the intervention’s programme theory [refer Ref 2]
- It would be helpful to give a rationale for why the Standards for Reporting Implementation Studies (StaRI) Checklist was selected as a framework for data extraction. Not all the studies reviewed will be implementation studies. Will this tool support extraction of the different types of data/information that will help explain the nuances of explaining how intentional rounding works and the contexts that influence this?

We hope these comments are helpful. In summary, our main point is that it would be helpful for this proposal for a realist synthesis to report and build on what is already known about the programme theory of intentional rounding. There are existing programme theories that have been refined through empirical evaluation, these should be reported and discussed and the review protocol designed to build on these. We think these revisions are necessary for the paper to be scientifically valid and therefore have no alternative than to select the publisher’s ‘Not approved’ status.

Is the study design appropriate for the research question?

Yes

Is the rationale for, and objectives of, the study clearly described?

Partly

Are sufficient details of the methods provided to allow replication by others?

No

Are the datasets clearly presented in a useable and accessible format?

Not applicable

Reviewer Expertise:

Nursing, realist synthesis and evaluation, intentional rounding, complex interventions

We confirm that we have read this submission and believe that we have an appropriate level of expertise to state that we do not consider it to be of an acceptable scientific standard, for reasons outlined above.

## References

[1] : Intentional rounding: a realist evaluation using case studies in acute and care of older people hospital wards. *BMC Health Serv Res* .2023;23(1) : 10.1186/s12913-023-10358-1 1341 10.1186/s12913-023-10358-1 38042788 PMC10693126

[2] : Evidence-Based Policy:A Realist Perspective. *Sage Publications Inc., London* .2006;

# Reviewer report: Reviewer response for version 1

**DOI:** 10.21956/hrbopenres.15090.r38009

**Published:** 2024-03-07

**Authors:** Sonya Kowalski

**Affiliations:** 1 University of Detroit Mercy, Detroit, Michigan, USA

This article is a realist synthesis research plan to investigate the contextual factors that impact the success or failure of intentional rounding (IR) strategies on inpatient fall rates. Cost factors are included in the synthesis plan.  

 The rationale for the study, to fill an information gap related to specific contextual factors that contribute to efficacy of IR strategies, is clear. The objectives are clearly written and included in the search and evaluation strategies. 

 The realist synthesis methodology is appropriate for the study. 

 The search strategy; inclusion/exclusion criteria; selection and appraisal; data extraction; data synthesis and analysis; theory refinement with stakeholders are clear. 

 The article is well written and the work is relevant to cost-effective patient safety.

Is the study design appropriate for the research question?

Yes

Is the rationale for, and objectives of, the study clearly described?

Yes

Are sufficient details of the methods provided to allow replication by others?

Yes

Are the datasets clearly presented in a useable and accessible format?

Not applicable

Reviewer Expertise:

Fall Risk Prevention Strategies

I confirm that I have read this submission and believe that I have an appropriate level of expertise to confirm that it is of an acceptable scientific standard.

# Response

**Published:** 2024-03-08

**Authors:** Aileen Hetherton

**Affiliations:** School of Post Graduate Studie, Royal College of Surgeons in Ireland Faculty of Sports and Exercise Medicine, Dublin, Leinster, Ireland

Dear Professor Kowalski, 

 Thank you for reviewing the article Contextual factors and intentional rounding in acute hospitals: understanding what works, for whom, in what settings: a realist synthesis protocol

 We are delighted to have received approval status from you and thank you for your commentary on our published work. 

 Kind Regards

 Aileen Hetherton

# Reviewer report: Reviewer response for version 1

**DOI:** 10.21956/hrbopenres.15090.r38011

**Published:** 2024-02-28

**Authors:** Amy Drahota, Leah Fullegar

**Affiliations:** 1 University of Portsmouth, Portsmouth, England, UK

Overall, this was a clearly written paper outlining the protocol for a realist synthesis on the use of intentional rounding in acute hospitals. The realist synthesis methodology is well referenced in the protocol; This is an appropriate method for the research topic. The inclusion of stage 6 (theory refinement with stakeholders) is a key stage in realist methodologies and good to see in the plan. We have a few key aspects of feedback for consideration, itemised below: 

**Introduction:**

 1. Paragraph 3. Maybe worth highlighting where the costs/figures being discussed are associated with all falls (including community-based falls), and the relationship of this to in-patient falls, given the focus of the review is on preventing hospital-based falls.

 2. Paragraph 4. There has been updated world guidelines published in Age & Ageing (Ref [1]) that might be beneficial to cite where NICE 2013 are cited.

 3. Paragraph 4. It would be worth citing the Cochrane review on preventing falls in hospitals, which is more up-to-date than the AHRQ 2013 (Ref [2]).

 4. Paragraph 8. Could perhaps signpost here that this publication sets out the protocol for the realist synthesis, and the second phase will be set out in future publications. Later on in the paper (see comment 11 below) it sometimes becomes confusing when the second phase is discussed within the realist synthesis methods.

**Rationale and existing reviews:**

 5. The rationale for the review could be further strengthened to explain how this review relates to Sims et al (2018) and explicitly indicate the gap in knowledge that necessitates the proposed review. By the end of the report it becomes clearer that this is an update of Sims (2018), however the paper would benefit from explaining this earlier on (and why there’s a need for an update), and to explain any ways in which this realist synthesis differs.

 6. Where it is stated that there has been no study in Ireland, is that in reference to primary evidence or to a realist synthesis having not yet been conducted in Ireland? Does that mean that this review is only going to focus on Irish primary research evidence, or that having the review undertaken in Ireland is going to shape the findings in some way that’s important? It’s currently a bit tricky to follow the line of argument as to why no study having yet been conducted in Ireland justifies this new review. It feels like perhaps this is a justification for the planned realist evaluation that is to follow in phase 2, rather than a justification for the synthesis, but it’s currently unclear.

**Realist synthesis methods:**

 7. The line of argument around assessing resources and cost is a bit difficult to follow in this section. Some of the writing and referencing appears to be more related to phase 2 (the realist evaluation) rather than this realist synthesis. We suggest this is reviewed to focus more specifically on the incorporation of economic data into the realist synthesis.

**Research aim and objectives:**

 8. The part of the aim on “promote stakeholder engagement in IR” comes as a bit of a surprise, having read the introduction/rationale, as stakeholder engagement isn’t explicitly mentioned up until this point, so it’s unclear what is being referred to here.

 9. The objectives appear clear, however objective 5 does not seem to relate to the aim; would it be worth revising the aim to incorporate the issue of resource use/costs?

**Stage one: clarifying scope and developing an initial programme theory**

 10. The stages of the review seem logical and fit with realist synthesis theory, however some clarity and reorganisation may be helpful in the protocol. Much of the information under the stage one heading describes the search strategy (snowballing) rather than explaining how the IPT will be developed and used. This section also states that the authors will be using the same search strategy as the existing realist synthesis on this topic, indicating they are aiming to update the existing review. This could do with clarifying earlier (see feedback point 5).

 11. It’s slightly confusing to discuss the two stages in this section. Does the *synthesis* have two phases (as stated) or is it that the overall project has two phases (i.e. a realist synthesis and realist evaluation)? It might be clearer simply to focus on stage one (the synthesis) here in this methods section, rather than referring to later stages of the project.

 12. Reference is made here to the inclusion/exclusion criteria being necessarily broad, however it is not yet clearly articulated what those eligibility criteria are. Consider where best to present the information on eligibility criteria (see comment 10 above).

 13. Will the initial programme theory be adapted from Sims et al (2018) or developed from scratch?

**Stage 2: search strategy**

 14. The inclusion and exclusion criteria do not include any mention of stakeholder engagement, patient safety outcomes, falls, or cost effectiveness – all of which have been discussed as focal points for this review. It would be helpful to clarify what is being investigated. Are there any limits placed on country, and dates?

**Stage 4: data extraction**

 15. The data extraction section states that the programme theories established in the existing realist review will be used but does not state how, this might fit better under Stage 1.

 16. This section also states only studies from 2016 to 2022 will be included, this information should be with the inclusion and exclusion criteria. The justification for this should also be clarified. Is it necessary to state 2022 as the upper date? What if newer research is discovered during the review process – will this not be eligible?

 To summarise, this was an enjoyable protocol to read. We suggest it could be improved further by strengthening the rationale, looking at the organisation of where content is being presented and enhancing the clarity around the eligibility criteria. We wish you well with this interesting and important research.

Is the study design appropriate for the research question?

Yes

Is the rationale for, and objectives of, the study clearly described?

Partly

Are sufficient details of the methods provided to allow replication by others?

Partly

Are the datasets clearly presented in a useable and accessible format?

Not applicable

Reviewer Expertise:

falls prevention; health & care of older adults; systematic reviews; realist synthesis.

We confirm that we have read this submission and believe that we have an appropriate level of expertise to confirm that it is of an acceptable scientific standard, however we have significant reservations, as outlined above.

## References

[1] : World guidelines for falls prevention and management for older adults: a global initiative. *Age Ageing* .2022;51(9) : 10.1093/ageing/afac205 10.1093/ageing/afac205 36178003 PMC9523684

[2] : Interventions for preventing falls in older people in care facilities and hospitals. *Cochrane Database Syst Rev* .2018;9(9) : 10.1002/14651858.CD005465.pub4 CD005465 10.1002/14651858.CD005465.pub4 30191554 PMC6148705

# Response

**Published:** 2024-04-24

**Authors:** Aileen Hetherton

**Affiliations:** School of Post Graduate Studie, Royal College of Surgeons in Ireland Faculty of Sports and Exercise Medicine, Dublin, Leinster, Ireland

We wish to thank you for your very thorough and insightful feedback on this protocol paper. 

 We have taken your sixteen points in to consideration and made amendments accordingly. 

 Your feedback and suggestions have been greatly appreciated and we hope the second version has addressed the concerns that you have highlighted. 

 We welcome any further feedback on this paper to ensure it is valid and will inform a scientifically sound realist synthesis study. 

 Once again thank you
